# QuickStats

**Published:** 2013-11-08

**Authors:** Arialdi Minino

**Figure:**
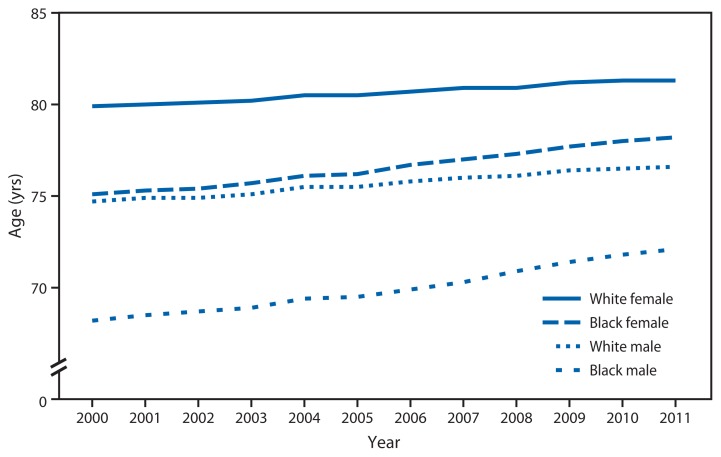
Life Expectancy at Birth, by Sex and Black or White Race — National Vital Statistics System, United States, 2000–2011^*^ ^*^ Data for 2011 are preliminary.

In 2011, life expectancy at birth for the overall U.S. population was 78.7 years. From 2000 to 2011, gains in life expectancy varied by race and sex, with the largest increase (5.7%) among black males, to 72.1 years. Life expectancy increased 4.1% among black females, to 78.2 years; 2.5% among white males, to 76.6 years; and 1.8% among white females, to 81.3 years.

**Sources:** Hoyert DL, Xu J. Deaths: preliminary data for 2011. Nat Vital Stat Rep 2012;61(6).

Murphy SL, Xu J, Kochanek KD. Deaths: final data for 2010. Nat Vital Stat Rep 2013;61(4).

